# Unlocking growth potential in *Halomonas bluephagenesis* for enhanced PHA production with sulfate ions

**DOI:** 10.1093/jimb/kuae013

**Published:** 2024-04-17

**Authors:** Fuwei Yao, Kai Yuan, Weiqiang Zhou, Weitao Tang, Tang Tang, Xiaofan Yang, Haijun Liu, Fangliang Li, Qing Xu, Chao Peng

**Affiliations:** School of food science and pharmaceutical engineering, Nanjing Normal University (NNU), Nanjing, 210023, China; Biotechnology Center, COFCO Nutrition and Health Research Institute Co., Ltd., Beijing, 102209, China; Biotechnology Center, COFCO Nutrition and Health Research Institute Co., Ltd., Beijing, 102209, China; COFCO Bio-Chemical Energy (Yushu) Co., Ltd., COFCO Biotechnology Co., Ltd., Changchun, 130400, China; Biotechnology Center, COFCO Nutrition and Health Research Institute Co., Ltd., Beijing, 102209, China; COFCO Bio-Chemical Energy (Yushu) Co., Ltd., COFCO Biotechnology Co., Ltd., Changchun, 130400, China; Biotechnology Center, COFCO Nutrition and Health Research Institute Co., Ltd., Beijing, 102209, China; COFCO Bio-Chemical Energy (Yushu) Co., Ltd., COFCO Biotechnology Co., Ltd., Changchun, 130400, China; Biotechnology Center, COFCO Nutrition and Health Research Institute Co., Ltd., Beijing, 102209, China; COFCO Bio-Chemical Energy (Yushu) Co., Ltd., COFCO Biotechnology Co., Ltd., Changchun, 130400, China; Biotechnology Center, COFCO Nutrition and Health Research Institute Co., Ltd., Beijing, 102209, China; COFCO Bio-Chemical Energy (Yushu) Co., Ltd., COFCO Biotechnology Co., Ltd., Changchun, 130400, China; Biotechnology Center, COFCO Nutrition and Health Research Institute Co., Ltd., Beijing, 102209, China; COFCO Bio-Chemical Energy (Yushu) Co., Ltd., COFCO Biotechnology Co., Ltd., Changchun, 130400, China; Biotechnology Center, COFCO Nutrition and Health Research Institute Co., Ltd., Beijing, 102209, China; COFCO Bio-Chemical Energy (Yushu) Co., Ltd., COFCO Biotechnology Co., Ltd., Changchun, 130400, China; School of food science and pharmaceutical engineering, Nanjing Normal University (NNU), Nanjing, 210023, China; Biotechnology Center, COFCO Nutrition and Health Research Institute Co., Ltd., Beijing, 102209, China

**Keywords:** *Halomonas bluephagenesis*, Polyhydroxyalkanoate, Sulfate ions, Fermentation scale-up

## Abstract

The mutant strain *Halomonas bluephagenesis* (TDH4A1B5P) was found to produce PHA under low-salt, non-sterile conditions, but the yield was low. To improve the yield, different nitrogen sources were tested. It was discovered that urea was the most effective nitrogen source for promoting growth during the stable stage, while ammonium sulfate was used during the logarithmic stage. The growth time of *H. bluephagenesis* (TDH4A1B5P) and its PHA content were significantly prolonged by the presence of sulfate ions. After 64 hr in a 5-L bioreactor supplemented with sulfate ions, the dry cell weight (DCW) of *H. bluephagenesis* weighed 132 g/L and had a PHA content of 82%. To promote the growth and PHA accumulation of *H. bluephagenesis* (TDH4A1B5P), a feeding regimen supplemented with nitrogen sources and sulfate ions with ammonium sodium sulfate was established in this study. The DCW was 124 g/L, and the PHA content accounted for 82.3% (w/w) of the DCW, resulting in a PHA yield of 101 g/L in a 30-L bioreactor using the optimized culture strategy. In conclusion, stimulating *H. bluephagenesis* (TDH4A1B5P) to produce PHA is a feasible and suitable strategy for all *H. bluephagenesis*.

## Graphical Abstract

**Graphical Abstract:**
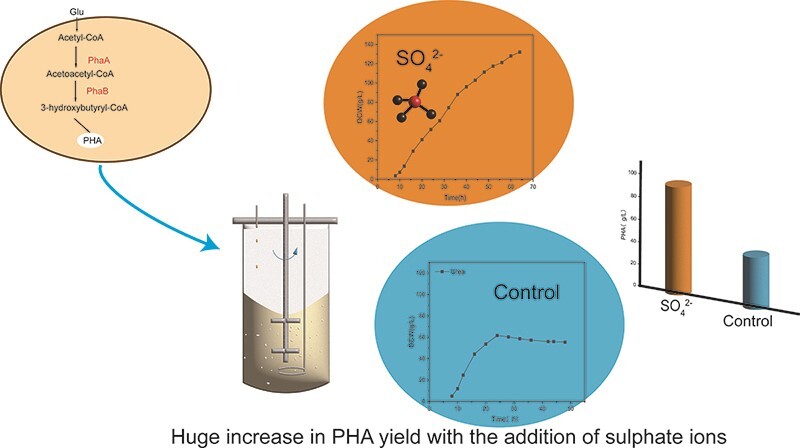

## Introduction

Traditional plastics have been widely used due to their simplicity, structural stability, and broad applicability in manufacturing various products for our daily lives with plastic production reaching 380 million metric tons in 2018 (Smith & Brisman, 2021; Stegmann et al., 2022). Traditional plastics are derived from non-renewable fossil fuels and are structurally stable and difficult to degrade down in the natural environment. Therefore, there is an urgent need to develop environmentally friendly and biodegradable alternatives to traditional plastics to minimize reliance on petroleum-based synthetic plastics. A variety of renewable plastics have been developed, such as PLA (polylactic acid) (Jambunathan & Zhang, 2016), (polybutylene adipate terephthalate) (Ferreira et al., 2019), and PHA (polyhydroxyalkanoates) (Yean et al., 2017). Polyhydroxyalkanoates, as a newly emerging bioplastic in recent years, possess significant developmental prospects due to their favorable physicochemical properties and excellent biodegradability (Sharma et al., 2021; Koller & Mukherjee, 2022). Polyhydroxyalkanoates is synthesized intracellularly as granules by prokaryotic organisms, serve as reserves of carbon and energy reserves (Madison & Huisman, 1999). They possess the properties of being biodegradable, renewable, and biocompatible (Muhammadi et al., 2015). The biodegradation of PHA can occur under natural conditions, breaking down into carbon dioxide and water (Muhammadi et al., 2015). Inside cells, PHA can protect bacteria from sudden osmotic pressure changes and external environmental shocks (Sedlacek et al., 2019). Additionally, PHA is a strategy employed by some microorganisms to cope with high osmotic pressures (Obruca et al., 2017). Microbes that synthesize PHA tend to do so prolifically when in an environment that is rich in carbon sources but deficient in other nutrients (such as nitrogen and phosphorus) (Steinbüchel, 1991). The biodegradability and structural diversity of PHA pave the way for a broader range of applications, such as in packaging materials and the medical field (heart valve prostheses, sutures, etc.) (Chen, 2009).

Although PHAoffer the advantages of being renewable and biodegradable, their high production cost—three to four times that of petroleum-based plastics—is a significant barrier to their commercialization (Bhola et al., 2021; Ye et al., 2018; Wang et al., 2022). While many microorganisms are capable of synthesizing PHAs, genetically engineered bacteria are frequently used for scaled industrial production (Ren et al., 2018). Species such as *Halomonas bluephagenesis* TD01 *Bacillus megaterium* (Findlay & White, 1983), and *Cupriavidus necator* (Schlegel et al., 1961; Morlino et al., 2023) have shown substantial developmental potential. Notably, *H. bluephagenesis* can accumulate PHA in excess of 70% of its dry cell weight (DCW). Based on the development of next generation industrial biotechnology (Chen et al., 2020), *H. bluephagenesis* uses non-sterile culture to reduce the production cost of PHA. To further reduce the cost of producing PHA for *H. bluephagenesis*, researchers have explored low-cost substrate fermentation models utilizing substrates such as xylose (Tan et al., 2022), starch (Lin et al., 2021; Liu et al., 2024), and food waste (Ji et al., 2023), with notable success. Inexpensive corn steep powder (CSP), a by-product rich in amino acids, microbes, and mineral elements from starch processing, has been demonstrated to serve effectively in culturing *H. bluephagenesis*, substituting for costlier nitrogen sources such as yeast extract and peptone (Ye et al., 2018). Some researchers have found that *H. bluephagenesis TD01* mutant cells lacking the *phaP* gene have a reduced number of PHA granules and an increase in granule size, with a single PHA granule sometimes being as large as the cell that contains it. Larger PHA granules facilitate purification and recovery (Shen et al., 2019). Additionally, a high cell density *H. bluephagenesis* TDHCD-R3 strain was created using a genome-wide random mutagenesis system that relies on an error-prone DNA polymerase III ε subunit. By overexpressing the optimized *phaCAB* operon, it attained a dry weight of over 90 g/L, with more than 79% of that weight being PHA (Ren et al., 2018). Studies have shown that strains of *H. bluephagenesis* with bacterial outer membrane defects were able to adapt to lower salt concentrations and exhibited characteristics such as greater oxygen uptake and increased cellular permeability (Wang et al., 2021). Various genetic manipulations have rendered *H. bluephagenesis* as a suitable candidate for PHA production.

*Halomonas bluephagenesis* produces PHA through a continuous feeding fermentation process, where the elemental content of different supplementation stages is adjusted to achieve a nitrogen-limited culture and promote PHA accumulation (Ye et al., 2018). While nitrogen limitation positively affects PHA accumulation, it greatly impairs cell growth and can shorten the logarithmic growth phase (Senez, 1962). Therefore, it is difficult for nitrogen-limited cultures to achieve both high PHA content and high DCW simultaneously. The growth of *H. bluephagenesis* and the accumulation of PHA is significantly influenced by the type of nitrogen source. This study uses *H. bluephagenesis* (TDH4A1B5P) as the host and improves its ability to produce PHA by at least 40% through adjustments to the feed medium, compared with existing technologies. The study investigates the effect of ammonium sulfate on the growth of *H. bluephagenesis* (TDH4A1B5P) during the feeding process and analyzes the specific impact of sulfate and ammonium ions, as well as sulfate ions alone, on the growth and accumulation of PHA in *H. bluephagenesis* (TDH4A1B5P). The most suitable cultivation method will be selected to scale up experiments and promote the industrial production of PHA.

## Materials and Methods

### Strains

*Halomonas bluephagenesis* (TDH4A1B5P) is provided by Tsinghua University. The strain was generated through ARTP mutagenesis to produce a low-salt-tolerant strain. The *PhaCAB* gene was then introduced into the strain using genetic engineering techniques.

### Shake Flask Cultivation

The bacteria were first isolated from the glycerol storage solution at −80 °C and then plated onto 15 LB agar (Luria–Bertani medium with 15 g/L NaCl) and revived at 37 °C for 24 hr. Subsequently, a single colony is picked from the 15 LB plate and inoculated into a 50 mL flask containing 20 mL of 15 LB liquid medium, then cultured at 37 °C with shaking at 200 rpm for 12 hr to obtain the primary seed culture. This primary seed culture is then inoculated at 1% (v/v) into a 500 mL flask containing 50 mL of 15 LB liquid medium, and incubated under the same conditions to achieve the secondary seed culture. Once the cell density reaches an OD600 of 4–5, the secondary seed culture is inoculated at 10% (v/v) into a 500 mL flask with 100 mL of 15MMG medium. The composition of this medium is as follows (g/L): NaCl 15, glucose 20, Urea 4, MgSO_4_ 0.2, KH_2_PO_4_ 3.7, NaH_2_PO_4_·12H_2_O 7.2, trace elements solution III, and trace elements solution IV (Ren et al., 2018).

### Cultivation Conditions and Feed Solution in 5 L and 30-L Bioreactors

After shake flask cultivation to obtain a secondary seed culture with an OD_600_ of 4–5, 10% (v/v) of this culture is transferred to inoculate 2.5 L of 15MMG medium in a 5-L bioreactor (Sartorius, Germany). This 5-L bioreactor serves as the seed culture for a 30-L bioreactor, where large-scale fermentation is typically initiated with a 10% (v/v) inoculum. During fermentation, air is sparged at a maximum rate of 1 VVM (air volume/culture volume/minute). After shake flask cultivation to produce a level two seed culture with an OD_600_ of 4–5, this culture is inoculated at a 10% (v/v) ratio into a 5-L bioreactor containing 2.5 L of 15MMG medium. Similarly, a 30-L bioreactor (Applikon, The Kingdom of the Netherlands) is inoculated proportionally based on volume scaling. A 10% (v/v) inoculum is commonly used for large-scale fermentations. During fermentation, stirring at less than 800 rpm is coupled with a maximum air sparge rate of 1 VVM (air volume/culture volume/minute). pH of the fermentation medium is maintained at 8.5 using 5 M NaOH. The optimized feeding strategy is partitioned into two stages: feed solution I and feed solution II. Feed solution I started when the glucose in the bioreactor was less than 10 g/L (about 8 hr from the start of fermentation)and feed solution II started after feed solution I was completed (about 20 hr from the start of fermentation). The whole fermentation was performed at 37 °C under open and non-sterilized conditions. Feed solution after optimization is suitable for both 5-L and 30-L bioreactors amplified according to the scale-up volume. Throughout the fermentation process, glucose concentration in the broth is maintained at approximately 10–20 g/L. During this process, samples are taken to measure the DCW and PHA contents of the culture.

### Analytical Methods

Cell growth was assessed utilizing a UV-Vis spectrophotometer (Biochrom, UK) at an optical density of 600 nm (OD_600_) after apt dilution to ensure that the initial OD_600_ was maintained between 0.3 and 0.8. Samples were collected from the culture every 2 hr, and the residual glucose concentration was immediately determined using a glucose analyzer (SBA-40E biosensor, Shandong Academy of Sciences, China). The DCW and PHA content of the culture were analyzed by sampling every 4 hr.

Harvest 30 mL of cell culture from shaker flasks or bioreactors, use a centrifuge (USA Thermo) at room temperature (25 °C) at 8000 rpm for 10 min. After centrifugation, wash the cells once with distilled water. Freeze the washed cell pellet at −80 °C for 24 hr, and then freeze dry it in a freeze dryer for 32–40 hr (USA GOLD SIM). Use the lyophilized biomass to measure the DCW.

To determine PHA content, lyse 30–40 mg of lyophilized cell powder at 100 °C in a decane solution composed of 2 mL of chloroform and 2 mL of methanolysis solution (97 wt% methanol, 3 wt% H_2_SO₄, and 1 g/L benzoic acid) for 4 hr. Then, after cooling to room temperature (25 °C), add 1 mL of water to extract and separate phases. The PHA content in the denser phase was analyzed by gas chromatography (Agilent, USA) with 35 mg of high-purity 3-hydroxybutyrate from Sigma–Aldrich as the standard (Tan et al., 2011).

## Results and Discussion

### *Halomonas Bluephagenesis* (TDH4A1B5P) Basic Growth Conditions

**Fig. 1.**
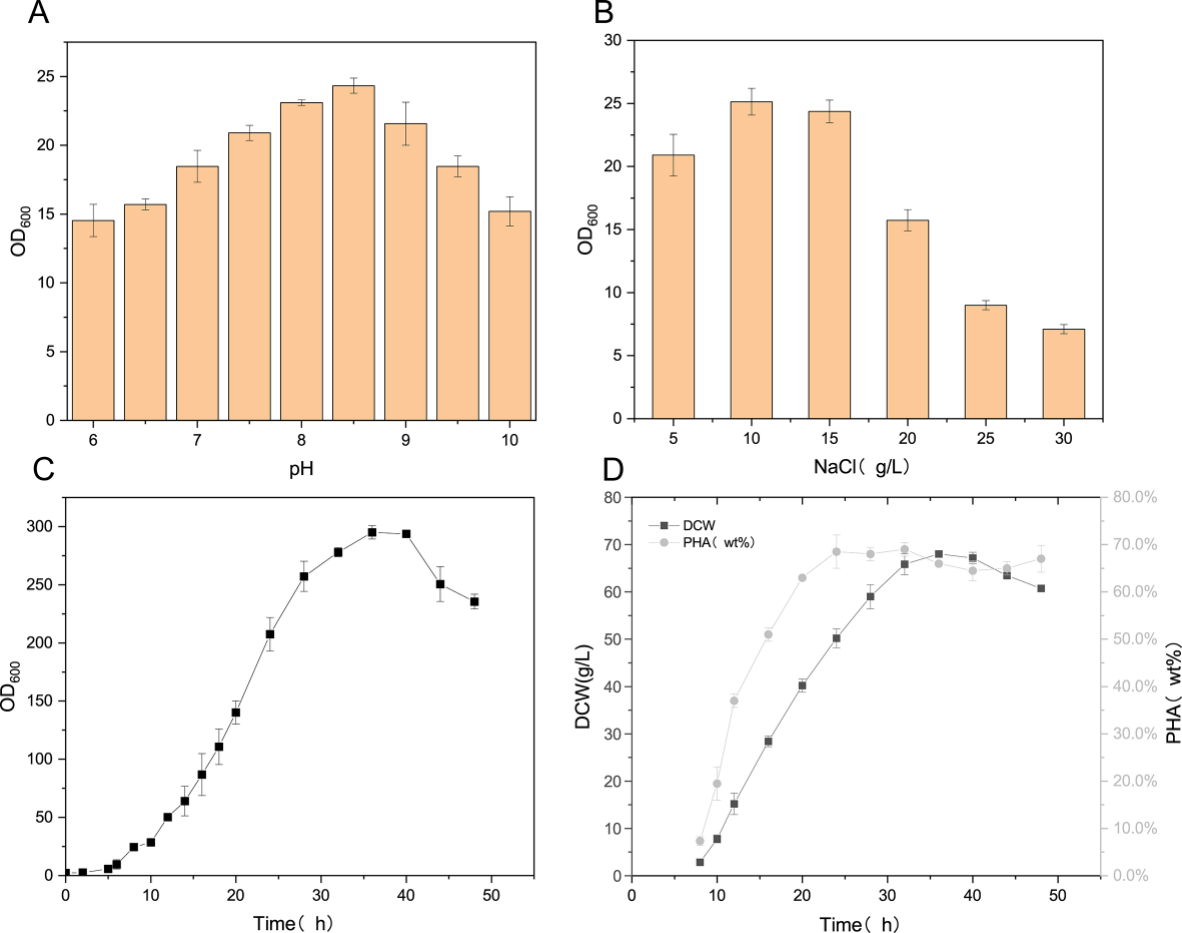
Basic growth conditions of *H. bluephagenesis* (TDH4A1B5P). (A) Growth of *H. bluephagenesis* (TDH4A1B5P) at different pH (OD600). (B) Growth of *H. bluephagenesis* (TDH4A1B5P) at different NaCl concentrations (OD600). (C) The changes in OD600 in a 5-L bioreactor. (D) The changes in dry cell weight (DCW) and PHA content in a 5-L bioreactor.

According to Fig. 1(A, B) show that the optimal growth conditions for this strain are at a pH of 8–9 with a NaCl concentration of 10–15 g/L. The optimal cultivation conditions for *H. bluephagenesis* (TDH4A1B5P) were identified as pH 8.5 with 15 g/L NaCl, taking into account the dilution effect of feeding on the NaCl concentration in the solution. This finding is consistent with relevant reports (Zhang et al., 2023). *Halomonas bluephagenesis* has a well-established culture protocol that uses corn syrup meal, urea, and ammonium sulfate as nitrogen sources and glucose as a carbon source. Figure 1(C, D) demonstrate that *H. bluephagenesis* (TDH4A1B5P) rapidly accumulates PHA from 6% to 70% within 8–24 hr of cultivation using the same medium for PHA fermentation. However, the cell biomass OD600 and the rate of DCW accumulation are significantly lower than that of PHA accumulation during this period. After 24 hr of cultivation, the cells PHA accumulation at 70%. No further increase in PHA content is observed as the majority of nutrients are metabolized through respiration instead of being channeled toward PHA accumulation. This leads to a slow increase in DCW and an extremely slow efficiency of PHA production after the 24-hr fermentation period. Therefore, optimizing the medium to prolong the growth period of *H. bluephagenesis* (TDH4A1B5P) is particularly important for obtaining higher PHA yields.

### The Influence of Different Nitrogen Sources on the Production of PHA By *H. Bluephagenesis* (TDH4A1B5P)

**Fig. 2.**
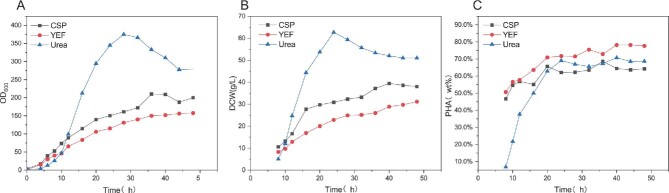
The impact of various single nitrogen sources on PHA production by *H. bluephagenesis* (TDH4A1B5P) during the fermentation process. (A–C) Cell growth (OD600), DCW, PHA content. Corn steep powder (CSP, circle), yeast extract fermentation (YEF, circle), urea (triangle), and ammonium sulfate (AMS, triangle).

Nitrogen source limitation is a common production strategy for PHA accumulation, yet nitrogen deficiency can affect bacterial cell growth (Meng et al., 2009). Figure 2 shows, during the cell retardation period (0–8 hr of fermentation), strains cultured with CSP and yeast extract fermentation (YEF) as the sole nitrogen sources exhibited rapid PHA accumulation, reaching 51% and 47%, respectively. In contrast, strains cultured with urea as the sole nitrogen source only accumulated 6.7% PHA. After 10 hr of fermentation, during the logarithmic growth phase, the growth rate and DCW accumulation rate of the strains cultured with urea were higher than those cultured with CSP and YEF. This suggests that the *H. bluephagenesis* (T01) cells cultured with CSP and YEF prematurely accumulated a large amount of PHA, which hindered the propagation and growth of the cells. Figure 2B and C demonstrate that, after approximately 28 hr of fermentation, the cells cultured with urea as the sole nitrogen source entered the decay phase. The DCW did not increase, and PHA stopped accumulating. The DCW of *H. bluephagenesis* (TDH4A1B5P) cultured with CSP and YEF grew slowly, but PHA production was almost stagnant. At 48 hr of fermentation, OD600 showed a decrease or almost no increase, and the fermentation ended. The DCW and PHA content of *H. bluephagenesis* (TDH4A1B5P) cultured with urea as the sole nitrogen source were the highest, at 62.67 g/L and 70 wt%, respectively. Cells fermented with YEF had the highest PHA content, at 78%, while those with CSP had a PHA content of 70%. However, the DCW performance of these two cultures was generally only 39.43 g/L and 31.14 g/L. It was found that culturing *H. bluephagenesis* (TDH4A1B5P) with a single nitrogen source did not provide any significant advantage in terms of DCW and PHA content. On the other hand, when CSP and YEF were used, cellular PHA was synthesized in large quantities during the delayed phase, which had an impact on the growth and reproduction of the bacteria. Thus, during the lag phase of cell culture, urea was utilized as the sole nitrogen source, while its nitrogen source was added after the lag phase to encourage PHA accumulation.

### Effect of Using Urea as the Sole Nitrogen Source in the Initial Medium and CSP, AMS, Urea as the Sole Unit Culture in the Replenishment Medium on the Production of PHA by *H. bluephagenesis* (TDH4A1B5P)

**Fig. 3.**
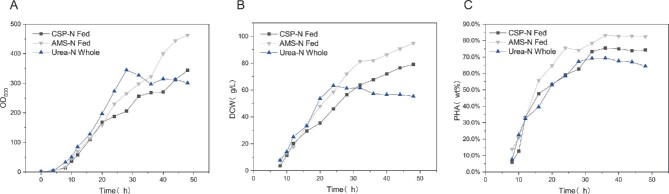
For the cultivation of *H. bluephagenesis* (TDH4A1B5P), urea was employed as the sole nitrogen source in the main culture medium, and other single nitrogen sources were used in the feeding medium. Figure (A–C) indicate cell growth (OD600), DCW, and PHA content. Corn steep powder as a source of nitrogen for feed solution (CSP-N Fed, black squares), urea as a source of nitrogen for feed solution (urea blue triangles), and ammonium sulfate as a source of nitrogen for feed solution (AMS-N Fed, gray triangles).

As shown in Fig. 3A–C, replenishment cultures with different single nitrogen sources were performed at the beginning of fermentation for 8 hr. Cells in the culture with urea as the sole unit in the replenishment phase at 28 hr of fermentation showed cell decay and PHA did not continue to accumulate, resulting in a final DCW of 63.3 g/L and 69.4% PHA content. Using CSP as the sole nitrogen source in the feeding phase, growth was maintained for 48 hr of fermentation and DCW reached 79.1 g/L at 48 hr. However, PHA content did not continue to accumulate at 32 hr of fermentation and PHA content was 74.3%. In the feeding regimen culture phase, when AMS was used as the sole nitrogen source, growth was also maintained for 48 hr and the growth rate was very high, reaching 94.9 g/L DCW at 48 hr. However, the PHA content did not accumulate at 36 hr, and the PHA content was 82.4%. Using urea as the sole nitrogen source in the initial medium and CSP or AMS as the sole nitrogen source in the feeding regimen culture phase prolonged the logarithmic growth rate of *H. bluephagenesis* (TDH4A1B5P), which did not reach the decay stage at 48 hr, allowing *H. bluephagenesis* (TDH4A1B5P) to obtain higher DCW. Corn steep powder is an industrial by-product resulting from the wet production of corn starch, which contains a high amount of sulfate ions. Thus, the positive stimulatory effect of CSP and AMS on PHA production in *H. bluephagenesis* (TDH4A1B5P) is likely due to the involvement of sulfate ions in cell growth metabolism. The complex composition of CSP may be the main reason for its difficulty in obtaining higher PHA content in cultured *H. bluephagenesis* (TDH4A1B5P).

### Effect of Sulfate Ion on Fermentation of *H. Bluephagenesis* (TDH4A1B5P)

**Fig. 4.**
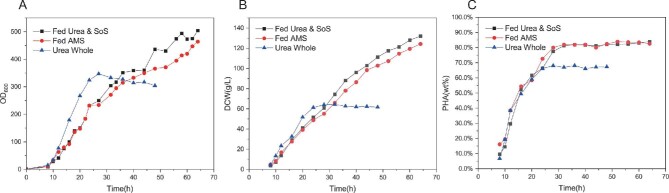
Throughout the study, urea served as the exclusive nitrogen source without additional sulfate ions (Urea Whole, triangles). The primary medium contained urea alone for nitrogen, while the feeding regimen culture phase utilized solely ammonium sulfate (Fed AMS, circles). During the corresponding replenishment, urea was used as the nitrogen source with sodium sulfate supplementation for sulfate ions (Fed Urea&SoS, squares). (A–C) Cell growth (OD600), DCW, PHA content.

As shown in Fig. 4B and C, *H. bluephagenesis* (TDH4A1B5P) cultured with urea as the sole nitrogen source achieved 132 g/L of DCW and 82% PHA production with the addition of sodium sulfate. The DCW and PHA content of *H. bluephagenesis* (TDH4A1B5P) reached 132 g/L and 82%, respectively, while the PHA yield reached 108.2 g/L. When grown in the main medium with urea as the sole nitrogen source and in the supplemented medium with ammonium sulfate as the sole nitrogen source, the strain achieved 124 g/L DCW, 82% PHA content, and 101.7 g/L PHA yield. In contrast, the culture with only urea added without sulfate ions had a DCW of only 64.33 g/L and a PHA content of 68.1%. This study demonstrated that sulfate ions have a positive effect on the growth and PHA accumulation of *H. bluephagenesis* (TDH4A1B5P). The addition of sulfate ions extended the logarithmic phase of *H. bluephagenesis* (TDH4A1B5P) and regulated its growth rate, while the logarithmic phase of *H. bluephagenesis* (TDH4A1B5P) cultured without sulfate ions was excessively rapid but shorter in duration. The presence of sulfate ions led to a 14% increase in PHA accumulation, which was directly related to the extension of the logarithmic phase of *H. bluephagenesis* (TDH4A1B5P). Figure 4A illustrates that the addition of sulfate ions prolonged the cell growth cycle from approximately 28 hr to about 64 hr, providing *H. bluephagenesis* (TDH4A1B5P) with ample time for biomass and PHA accumulation.

**Fig. 5.**
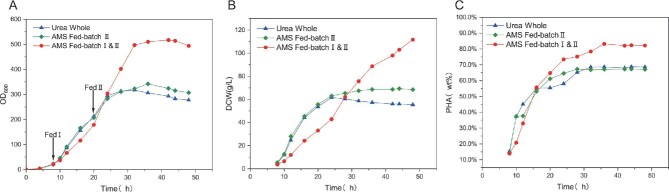
The whole process was carried out by replacing urea with ammonium sulfate in urea culture at different periods to investigate the effect of ammonium sulfate culture on PHA production of *H. bluephagenesis* (TDH4A1B5P) at different periods. Urea culture was used in the whole process (urea whole triangle), ammonium sulfate culture was used in both feed solution Ⅰ and feed solution Ⅱ (AMS Fed Ⅰ&Ⅱ, circle), and ammonium sulfate culture was used only in feed solution Ⅱ(AMS Fed Ⅰ, diamond). (A–C) Cell growth (OD600) and feed solution Ⅰ and Ⅱ periods, DCW, PHA content.

### Effects of Sulfate on *H. Bluephagenesis* (TDH4A1B5P) Stimulation At Different Periods and Concentrations

As depicted in Fig. 4B and C, the strain cultured with AMS in both the first and second supplementation stages reached a PHA content of 82.2% and a DCW of 111.67 g/L after 48 hr. The strain cultured with urea only before the second replenishment did not reach the same level of ammonium sulfate culture, which reached 68.4 g/L and 67.1% of DCW and PHA content at 48 hr. The DCW of the urea culture program improved slightly to 55 g/L, but the PHA content did not show significant improvement. This passage describes how sulfate ions stimulate *H. bluephagenesis* (TDH4A1B5P) to accumulate PHA, primarily during the logarithmic phase of growth. However, adding sulfate ions to the strain as it approaches the end of the logarithmic phase does not continue to stimulate growth or PHA accumulation. Additionally, the text should adhere to conventional academic structure and formatting, including consistent citation and footnote style. Finally, the passage should be free from grammatical errors, spelling mistakes, and punctuation errors. It is important to maintain a clear and objective tone, avoiding biased or figurative language. At the beginning of the logarithmic phase, *H. bluephagenesis* (TDH4A1B5P) effectively increased the DCW and PHA content of the strain. This increase may involve sulfate ions in the intracellular metabolism and synthesis of sulfur-containing proteins, promoting the growth of the bacterium and the accumulation of PHA. As the cells approached the end of the logarithmic phase, a large amount of intracellular PHA accumulated, leading to the inhibition of growth and metabolism of the bacterium. At the end of the logarithmic phase, the intracellular accumulation of PHA hindered the growth and metabolism of the bacterium. It was difficult to add sulfate ions at this point to metabolize them into sulfur-containing proteins or synthesize them with low efficiency, resulting in insignificant growth of *H. bluephagenesis* (TDH4A1B5P).

### Optimizing the Carbon-To-Nitrogen Ratio in the Feed Solution Ⅱ

**Fig. 6.**
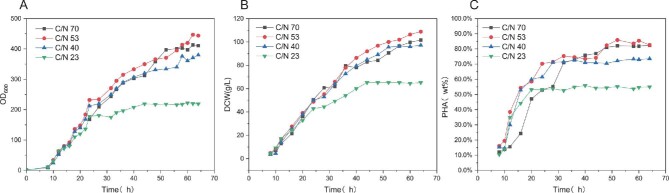
Effect of different carbon to nitrogen ratios in the second feed solution Ⅱ culture medium on PHA production by *H. bluephagenesis* (TDH4A1B5P). (A–C) Cell growth (OD600), DCW, PHA content.

The several studies have demonstrated that nitrogen deficiency halts microbial division, leading to the accumulation of lipids in non-dividing cells (Ratledge, 2002; Silva et al., 2017). It is observed in Fig. 6B and C) that *H. bluephagenesis* (TDH4A1B5P) cultured at a lower carbon to nitrogen (C:N) ratio (23:1) exhibited the lowest DCW and PHA content of 65 g/L and 55%, respectively. The DCW did not increase after 44 hr, and the cellular PHA content barely increased after reaching 553% at 20 hr. This may be due to the lower C:N ratio used for the production of PHA in Fed II, which was not employed in the fermentation of *H. bluephagenesis* (TDH4A1B5P). The lower C:N ratio may have caused *H. bluephagenesis* (TDH4A1B5P) to utilize glucose at a slower rate than nitrogen (N) utilization efficiency. This resulted in the accumulation of higher concentrations of N in the medium, which affected the growth and PHA accumulation of *H. bluephagenesis* (TDH4A1B5P). After 50 hr of fermentation, the medium-low C:N ratio (40:1) had a significant inhibitory effect. At the end of 64 hr of fermentation, the DCW stabilized at 97 g/L and the PHA stopped accumulating after 30 hr, with the PHA content remaining at 70%. This supports the conclusion that the growth and PHA accumulation of *H. bluephagenesis* (TDH4A1B5P) were inhibited due to the low carbon and nitrogen ratio (23:1). The insufficient utilization of nitrogen resulted in high nitrogen concentration in the medium, which further hindered the growth and PHA accumulation. An optimal medium-high C:N ratio of 57:1 consistently promoted stable DCW growth, reaching 108 g/L and 82% PHA content after 64 hr. However, the higher C:N ratio (70:1) resulted in a slightly lower growth rate of *H. bluephagenesis* (TDH4A1B5P) than the medium-high C:N ratio (57:1). This resulted in a DCW of 101 g/L at the end of the 64-hr fermentation. The PHA accumulation was not significantly affected by the C:N ratio, which may be attributed to the fact that *H. bluephagenesis* (TDH4A1B5P) is a versatile strain. *Halomonas bluephagenesis* (TDH4A1B5P) did not grow in the medium-high C:N ratio (57:1). However, PHA accumulation was not significantly affected by the end of the 64-hr fermentation. It is possible that *H. bluephagenesis* (TDH4A1B5P) consumed nitrogen at a higher rate than glucose, resulting in low nitrogen inhibition in the fermenter and limiting bacterial growth. However, the lower nitrogen concentration did not significantly affect PHA accumulation. The most suitable feed solution for *H. bluephagenesis* (TDH4A1B5P) was a C:N ratio of (57:1) in the second supplemented medium.

### Scale-Up of PHA Production in 30-L Fermenters

**Fig. 7.**
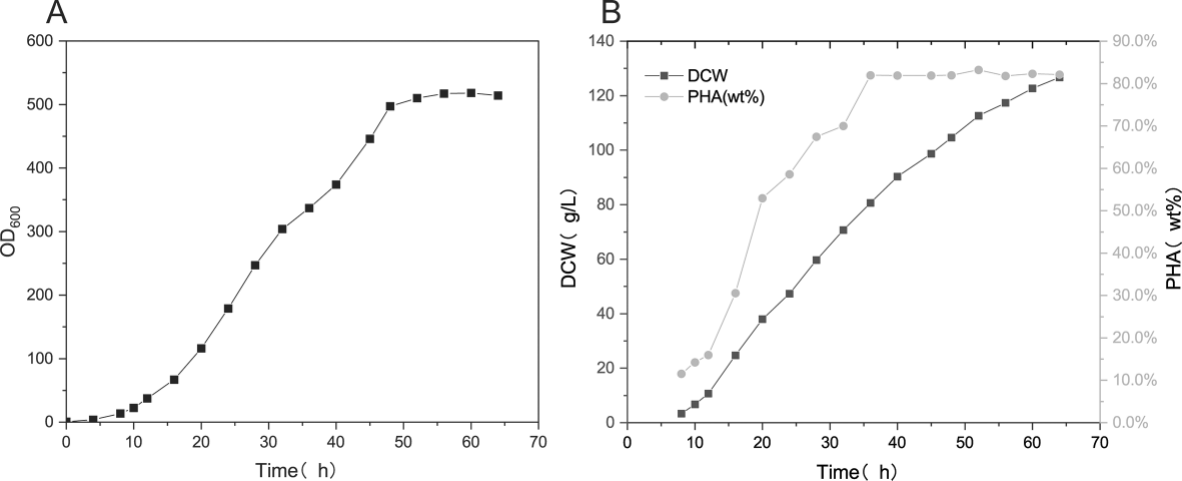
A feed solution utilizing sulfate ions was tested in a 30-L fermenter that maintained the same volumetric ventilation rate, number and shape of impeller blades, and impeller blade tip speed as the 5-L fermenter. (A) OD600, (B) DCW, PHA content.

Figure 7B shows that after 36 hr of fermentation, the PHA content reached 82%, which was maintained until the end of fermentation. Fermentation was stopped at 64 hr, and the DCW reached 126.7 g/L. After 36 hr of fermentation, the DCW continued to increase, but *H.luephagenesis* (TDH4A1B5P) PHA did not accumulate. The efficiency of PHA production was lower before 36 hr of fermentation than during the 36–64 hr period. The addition of sulfate ions to the feed solution prolonged the fermentation time, but improved the growth efficiency of PHA. The 30 L fermenter ultimately yielded 103.9 g/L of PHA. Although there was a 3% difference in DCW between the 30-L and 5-L bioreactors, there was no significant difference in PHA content, and the DCW fell within the experimental error. Addition of sulfate ions during the feeding regimen phase resulted in PHA yields in excess of 100 g/L for both the 5-L and 30-L bioreactors. This statement confirms that the feed solution of *H. bluephagenesis* (TDH4A1B5P) for PHA production, with the addition of sulfate ions at the replenishment stage, is suitable for industrial production.

## Discussion

In bacterial metabolism, sulfate ions are reduced via two principal pathways: assimilatory sulfate reduction (ASR) and dissimilatory sulfate reduction (DSR).

The DSR pathway begins with the transport of sulfate ions (SO_4_^2−^) into the cell via a sulfate transporter protein, located in the cell membrane. Once inside the cell, the sulfate is reduced to sulfite (SO_3_^2−^) by the enzymes ATP sulfurylase and APS reductase. The sulfite is then further reduced to hydrogen sulfide (H_2_S), through the action of sulfite reductase (Qian et al., 2019; Zhang et al., 2022). It is worth noting that during the cultivation of *H. bluephagenesis*, pungent H_2_S gas is not produced; DSR is not the process by which *H. bluephagenesis* reduces sulfate ions. Instead, *H. bluephagenesis* can utilize sulfate ions through the ASR pathway. This pathway involves a sequential conversion process: [sulfate → adenosine 5′-phosphosulfate (APS) → 3′-Phospho-5′-adenylyl sulfate → sulfite → sulfide] (Li et al., 2019). The sulfide is predominantly incorporated into cysteine. Cysteine is a crucial building block for protein synthesis and a precursor for methionine and other essential sulfur-containing biomolecules, which are necessary for various physiological and metabolic functions within organisms.

Feeding *H. bluephagenesis* with sulfate ions may lead to higher cell dry weight, potentially due to the biosynthesis of three key sulfur-containing biomolecules: the amino acids L-cysteine and methionine, and the tripeptide glutathione. These molecules, critical for various cellular functions, can contribute to biomass accumulation when sulfur is sufficiently available. The disulfide bonds formed by L-cysteine are essential for maintaining protein structure (Marino & Gladyshev, 2010), which is crucial for proteins to perform various physiological functions within the organism, such as participating in signal transduction, constructing structural proteins, and acting as enzymes (Giles et al., 2003; Pace & Weerapana, 2013). Methionine is not only vital for protein synthesis but is also the precursor of S-adenosylmethionine (SAMe). As the main methyl donor in organisms, SAMe plays an important role in methylation of DNA and histones (Brosnan et al., 2007), which can regulate gene expression, thereby influencing cell proliferation and differentiation (Aledo, 2019; Drazic et al., 2013). Glutathione is fundamental in protecting cells from oxidative stress damage and is crucial for maintaining the intracellular redox balance (Hatem et al., 2014). When cells are stimulated by growth factors, their internal redox status may change. Glutathione supports cell proliferation and survival by helping to cope with these changes (Grant et al., 1996).

## Conclusion

Historically, *H. bluephagenesis* has not been cultured with more than 100 g/L of DCW, and the analysis of the traditional medium formulation in this study showed that the addition of sulfate ions to the supplemented medium greatly increased the DCW and PHA content, with a DCW of 126 g/L and a PHA content of 82% achieved after 64 hr of fermentation in a 30-L fermenter, and a PHA yield of 103.9 g/L. This is a level never before achieved in *H. bluephagenesis* culture, and the addition of sulfate ions is not only applicable to *H. bluephagenesis* (TDH4A1B5P). The PHA production reached 104 g/L, which is a level of DCW never seen before in *H. bluephagenesis* culture. The feeding program involving the addition of sulfate ions was also effective for *H. bluephagenesis* (TD01), which achieved a high DCW with this approach. Feed solution with sulfate ions has allowed *H. bluephagenesis* to achieve significant yield increases for many chemical products produced in the field of synthetic biology.
